# YQHX Alleviates H/R-Induced Cardiomyocyte Apoptosis by Downregulating miR-1

**DOI:** 10.1155/2021/4852406

**Published:** 2021-11-02

**Authors:** Luandie Ge, Yaqi Fan, Lin Fu, Mengjiao Guo, Panxia Cao, Chaojie Peng, Linke Wu, Lihua Han, Hong Wu

**Affiliations:** ^1^Graduate School, Henan University of Chinese Medicine, Zhengzhou 450046, China; ^2^Institute of Cardiovascular Disease, Henan University of Chinese Medicine, Zhengzhou 450002, China; ^3^Laboratory of Cell Imaging, Henan University of Chinese Medicine, Zhengzhou 450002, China

## Abstract

Yiqi Huoxue granule (YQHX) inhibits cardiomyocyte apoptosis in myocardial ischemia-reperfusion injury (MIRI); however, the underlying mechanism is unknown. In this study, hypoxia-reoxygenation (H/R) models were established using rat myocardial primary cells and H9c2 cells, lactate dehydrogenase (LDH), and creatine kinase (CK) levels and cardiomyocyte apoptosis were determined. LDH release, CK activity, caspase-3 activation, mRNA and protein ratio of Bax/Bcl-2, and miR-1 expression were significantly higher (*p* < 0.01) in the H/R model of rat myocardial primary cells and H9c2 cells compared with the control group and was inhibited by YQHX treatment (*p* < 0.01 or *p* < 0.05). We also found that miR-1 overexpression could enhance apoptosis in cardiomyocytes, whereas apoptosis could be reduced by YQHX treatment (*p* < 0.01). In conclusion, YQHX alleviates H/R-induced cardiomyocyte apoptosis by inhibiting miR-1 expression, suggesting the potential of YQHX in preventing MIRI.

## 1. Introduction

Acute myocardial infarction (AMI) has emerged as one of the major diseases endangering human health and life [1]. In clinical practice, AMI is mainly treated by percutaneous coronary intervention or drug thrombolysis; however, upon rapid restoration of coronary blood supply, myocardial ischemia-reperfusion injury (MIRI) is inevitable [2–4]. Cardiomyocyte apoptosis is the main pathological basis of MIRI. Anticardiomyocyte apoptosis therapy can effectively alleviate the degree of MIRI and improve prognosis [5]. Commonly used drugs, such as calcium antagonists and *β*-receptor blockers are clinically effective against MIRI cardiomyocyte apoptosis; however, their curative effect is not ideal [6]. Therefore, developing effective anti-cardiomyocyte apoptosis drugs is of great clinical significance to improve the prognosis of AMI.

According to the theory of traditional Chinese medicine, “Qi deficiency” and “blood stasis” are the basic features of MIRI pathogenesis [7]. Interestingly, traditional Chinese medicines used to treat “Qi deficiency” and “blood stasis” in AMI have significantly alleviated MIRI [8, 9]. Yiqi Huoxue granule (YQHX), composed of ginseng, astragalus, safflower, and red peony, is a common prescription made by Professor Han Lihua for the clinical treatment of cardiovascular diseases. It is known for its Qi-invigorating and blood-activation properties leading to a definite curative effect [10, 11]. Previous studies have shown the anti-inflammatory properties of YQHX reducing calcium-ion and antioxidant damage [11–13], which are the main pathological factors of cardiomyocyte apoptosis in MIRI. YQHX has also been shown to alleviate H_2_O_2_-induced oxidative damage in cardiomyocytes and inhibit cardiomyocyte apoptosis by upregulating coupling protein 2 (UCP2) [14]. However, it remains unclear whether YQHX intervenes with cardiomyocyte apoptosis during MIRI.

miR-1 has been closely associated with ischemic heart disease, including myocardial infarction and cardiomyocyte apoptosis [15]. Its effect has been linked to the regulation of apoptosis-related genes Bax and Bcl-2 and been reported that downregulation of miR-1 improves MIRI and protects cardiac function [16]. Interestingly, several traditional Chinese medicine compounds and monomers have been found to exhibit anticardiomyocyte apoptosis effects via regulating miR-1. For example, Xinkang granules decreased myocardial injury in chronic heart failure by inhibiting miR-1 [17]. Likewise, monomers such as astragaloside IV and ligustrazine inhibit the apoptosis of ischemic myocardial cells [18, 19]. However, only a few studies have evaluated the intervention of traditional Chinese medicine in MIRI-induced myocardial cell apoptosis via miR-1 regulation. Importantly, the antioxidative damage effect of safflower extract hydroxysafflower yellow, a component of YQHX, is related to the inhibition of miR-1 expression [20]. Therefore, we speculated that YQHX could potentially regulate miR-1 expression.

In this study, primary rat cardiomyocytes and H9c2 cardiomyocytes were used to establish a hypoxia-reoxygenation (H/R) model to examine the intervention effect of YQHX. We found that YQHX could alleviate myocardial injury, reduce apoptosis, and regulate the expression of miR-1, thereby suggesting that miR-1 could be a potential therapeutic target for YQHX in the treatment of MIRI and other cardiovascular diseases.

## 2. Materials and Methods

### 2.1. Animals

38-week-old 30 male Sprague Dawley (SD) rats weighting 250–300 g (animal certificate number: 41003100006612; ethics approval number: PZ-HNSZY-2019-043) were purchased from the Henan Experimental Animal Center (Zhengzhou, China), housed at 25°C, subjected to a 12 h/12 h light/dark cycle. The experimental animal production license was SYXK (Yu) 2017–0001. All animal experiments followed the “Guidelines for the Feeding and Use of Experimental Animals” and were approved by the Ethics Committee of the Second Clinical Medical College of Henan University of Traditional Chinese Medicine (Zhengzhou, China).

### 2.2. Reagents and Drugs

Fetal bovine serum, trypsin (Israel Bioindustry Company, Beit-Haemek, Israel); collagenase 2, creatine kinase (CK), MTT kit (Solarbio, Beijing, China); lactate dehydrogenase (LDH) kit (Beyotime Biotechnology, Shanghai, China); and miRcute miRNA extraction and isolation kit (Tiangen Biotech, Beijing, China) were used in this study. First-strand cDNA of miRNA was synthesized using the stem-loop method (Sangon, Shanghai, China), anti-Bax antibody (ab53154), and anti-Bcl-2 antibody (ab196495) were purchased from Abcam (Cambridge, UK); anti-caspase-3 antibody (cat. no. 66470-2-Ig), anti-GAPDH antibody (cat. no. 10494-1-AP), and enzyme-labeled goat anti-rabbit secondary antibody (cat. no. SA00001-2) were obtained from Proteintech Group (Wuhan, China); BCA kit was purchased from Boster (Wuhan, China); ECL kit was from Thermo Fisher (Massachusetts, USA); Joklik was from Sigma (Missouri, USA); and laminin solution was from Roche (Shenzhen, China). YQHX, composed of astragalus (30 g), ginseng (15 g), safflower (15 g), and red peony (10 g), was purchased from Sichuan Neo-Green Pharmaceutical Technology Development Co., Ltd. (Sichuan, China). Batch numbers of single Chinese Medicines were as follows: astragalus (no. 17010092), ginseng (no. 17010055), red peony (no. 16060138), and safflower (no. 16080067). YQHX particles, crushed to powder, were mixed with calcium-free tabletop solution (NaCl 140 mmol/L, KCl 5 mmol/L, MgCl_2_ 1.2 mmol/L, D-glucose 2 mmol/L, HEPES 10 mmol/L, and pH 7.4) and dissolved by heating and ultrasound. After filtering and sterilizing using a 0.22 *μ*m membrane filter (Missouri, USA), a 200 mg/mL YQHX mother solution was prepared and stored at 4°C for later use.

### 2.3. Isolation and Culture of Rat Primary Cardiomyocytes

A 10 cm cell-culture dish containing Joklik was placed on ice. Heparin sodium (0.625 U/mL) was injected intraperitoneally in SD rats (0.1 mL/100 g). After 10 min, 20% urethane solution (1 g/kg) was injected into the abdominal cavity. Next, the thoracic cavity was opened, and the heart was resected and placed in a culture dish. After the heart was removed, the rat was immediately euthanized by cervical dislocation. Redundant tissue was stripped off to free the aorta. The heart was washed with Joklik perfusate at a constant temperature of 37°C. After 2-3 min, the perfusate was replaced with collagenase circulatory perfusion. After 30–45 min, the digestion was terminated following turbidity of the circulatory perfusate and hardness of the heart. During the entire perfusion process, a continuous perfusion of pure oxygen was ensured. Residual tissue was removed using a 100-mesh filter (Missouri, USA). To obtain the supernatant, the solution was centrifuged at 2000 rpm for 20 s at room temperature (RT). Separation was facilitated by the addition of gradient recalcification solution using natural sedimentation in a water bath at 37°C, and the process was carried out three times. Three hours before the separation of primary cardiomyocytes, 1 mL of M199+ medium was added to 4% laminin precoated 6-cm cell-culture dishes. After complete separation, the culture dishes were gently shaken. Culturing of primary cardiomyocytes was carried out in a constant temperature incubator at 37°C and 5% CO_2_ for 3 h. The original medium was discarded, and fresh M199+ medium was added. Cells were randomly labeled and divided into groups.

### 2.4. Preparation of an H/R Model of Rat Primary Cardiomyocytes and H9c2 Cells

H9c2 cells were purchased from Shanghai Cell Bank of Chinese Academy of Sciences (Shanghai, China). H9c2 cells or rat primary cardiomyocytes were seeded in 6-well plates and cultured until 90% confluence. Then, cells were randomly divided into 3 groups and subjected to the following interventions: (1) control group (cells were cultured in normal conditions without any treatment); (2) H/R group (cells were placed in a closed anoxic chamber for 3 h, the culture medium was changed, and the cells were cultured in 5% CO_2_ in an incubator for 0.5 h); (3) YQHX group (cells were pretreated with YQHX for 12 h prior to H/R treatment). Lastly, the cells were harvested for subsequent experiments.

### 2.5. Cell Transfection

H9c2 cells (1.5 × 10^5^ cells/well) were seeded into 6-well plates. At 50% confluency, the original medium was discarded, and the cells were washed with PBS. Then, DMEM containing 2% fetal bovine serum was added. NC mimics (sense: UUCUCCGAACGUGUCACGUTT, antisense: ACGUGACACGUUCGGAGAATT) or miR-1 mimics (sense: UGGAAUGUAAAGAAGUAUGUAU, antisense: ACACACUUCUUUACAUUCCAUU) were transferred using riboFECT™ CP transfection reagent (RiboBio, Guangzhou, China). Cells were harvested 48 h after transfection for the indicated experiments.

### 2.6. Cell Viability Assay

Primary cardiomyocytes were seeded in a 96-well plate at a density of 1.5 × 10^3^ cells/well. Next, different concentrations of YQHX were added. Experiments for each YQHX concentration were performed in triplicate. After culturing for 12 h, 10 *μ*L of 1.0 mg/mL MTT solution was added to each well. The plate was incubated at 37°C and 5% CO_2_ for 4 h and mixed by slow shaking for 10 min. The absorbance of the cells in the wells was measured at 490 nm using a microplate reader (Thermo Scientific, Massachusetts, USA). Similarly, H9c2 cells were seeded in 96-well plates at a density of 7 × 10^3^ cells/well and subjected to similar treatment as that used for the primary cardiomyocytes to determine cell viability.

### 2.7. Estimation of CK Activity in Rat Cardiomyocytes

Following the instructions of CK kit (Beijing, China), cells were detached and collected using a crude enzyme solution. Then, cells were crushed in an ice bath using ultrasonication and centrifuged at 1000 g and 4°C for 10 min. The supernatants collected were placed on ice. Next, 200 *μ*L of the supernatant, 450 *μ*L of working solution, and 350 *μ*L of H_2_O were added to a 1 mL cuvette. After thorough mixing, the absorbance of the reaction mixture was measured at 340 nm for 10 s. Subsequently, the cuvette containing the mixture was placed in a 37°C water bath for 3 min to dry the sample. Then, the absorbance of the reaction mixture was measured for 190 s at 340 nm, and CK activity was calculated according to the instructions in the kit.

### 2.8. LDH Detection

Cell culture medium was collected and centrifuged at 400 g and 4°C for 5 min. The supernatant was added to a 96-well plate (120 *μ*L/well), and the zero-setting group was established (i.e., 120 *μ*L fresh medium). Then, 60 *μ*L of LDH detection working solution was added to each well. After proper mixing, the mixture was incubated in the dark RT for 30 min, and the absorbance was measured at 450 nm using a microplate reader (Thermo Scientific, Massachusetts, USA).

### 2.9. qRT-PCR

To determine miR-1 expression levels, total miRNA was extracted from cells using the miRcute miRNA extraction and isolation kit. Reverse transcription was performed using an miRNA first-strand cDNA synthesis kit (cervical-loop method). To determine Bax and Bcl-2 mRNA levels, total RNA extracted from the cardiomyocytes was reverse transcribed into cDNA. Quantitative real-time PCR was performed using TB Green Premix Ex Taq GC (TaKaRa, Dalian, China). Samples were analyzed in triplicate and GAPDH (or U6) was used as the internal reference gene. The products were predenatured at 95°C for 30 s, denatured at 95°C for 5 s, annealed at 60°C for 30 s, and amplified for 36 cycles. After the reaction, Ct values were obtained determined using the 7500 software program. Data were exported for statistical analysis, and the 2^−△△Ct^ method was used for calculations. Primers that were used are shown in Table 1.

### 2.10. Western Blotting

Rat primary cardiomyocytes and H9c2 cells were lysed in RIPA lysis buffer, and protein concentrations were measured using the BCA method. Each sample was mixed with 5×loading buffer and heated at 100°C for 5 min. After 10% SDS-PAGE gel electrophoresis, protein samples were transferred onto PVDF membranes and blocked with 5% skimmed milk powder at RT for 1 h. Then, the membranes were incubated with 1 : 1000 anti-Bax, anti-Bcl-2, and 1 : 5000 anti-GAPDH primary antibodies at 37°C for 1.5 h or at 4°C overnight, washed with TBST, and incubated with secondary HRP-conjugated antibodies (diluted 1 : 1000), including anti-mouse or anti-rabbit antibodies for 1 h at RT. Next, membranes were washed three times with TBST solution for 10 min. PVDF membranes were placed in ECL solution, and images were acquired using a gel-imaging system. The gray values of the respective protein bands were analyzed using ImageJ software.

### 2.11. Statistical Analysis

All data are expressed as the mean ± standard deviation ($\bar{x}$ ± *s*). The statistically significant differences between groups were determined using one-way ANOVA and post hoc Tukey's test. *p* < 0.05 or *p* < 0.01 indicated that the difference was statistically significant.

## 3. Results

### 3.1. Screening for YQHX Cytotoxicity

**Figure 1:**
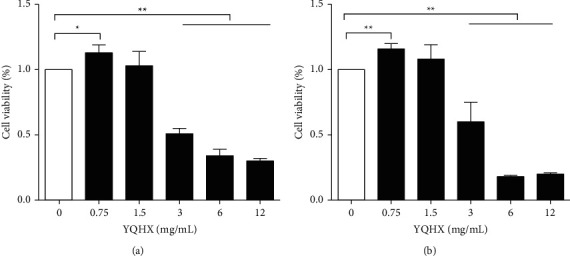
Cytotoxicity of YQHX. (a) Primary cardiomyocytes were incubated with different concentrations of YQHX (0.75, 1.5, 3, 6, and 12 mg/mL) for 12 h, and cell viability was determined using the MTT assay. (b) H9c2 cells were incubated with different concentrations of YQHX (0.75, 1.5, 3, 6, and 12 mg/mL) for 12 h, and cell viability was determined. Data are expressed as mean ± SD of three independent experiments. ^*∗*^*p* < 0.05 and ^*∗∗*^*p* < 0.01 were considered statistically significant.

According to the Chinese Pharmacopoeia [21], nontoxic drugs' high concentration can cause toxic reactions and damage human tissues and organs. Therefore, we should determine the nontoxic and effective drug concentrations. In *in vitro* studies performed using rat primary cardiomyocytes and H9c2 cells, the 0.75 mg/mL YQHX group exhibited an increase in cell viability compared with the control group (Figures 1(a) and 1(b)). However, when the concentration of YQHX was 1.5 mg/mL, no significant differences were observed between the two groups. Moreover, the cell viability decreased significantly in the treatment groups compared with the control group when the YQHX concentration was higher than 3 mg/mL.

### 3.2. YQHX Attenuated the Degree of Cell H/R Damage

**Figure 2:**
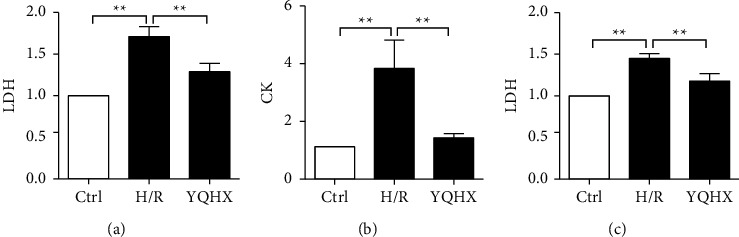
YQHX attenuates the extent of cell H/R damage. In the H/R groups, primary cardiomyocytes and H9c2 cells were placed in a hypoxic chamber for 3 h and then cultured for 0.5 h under normal conditions. In the YQHX group, cells were pretreated with YQHX for 3 h prior to H/R treatment. (a) LDH release in primary cardiomyocytes was determined by colorimetry. (b) CK activity in primary cardiomyocytes was estimated by colorimetry. (c) LDH release in H9c2 cells was estimated by colorimetry. Data are expressed as mean ± SD of three independent experiments. ^*∗∗*^*p* < 0.01 was considered statistically significant.

Based on the above results, 0.75 mg/mL of YQHX was determined as the optimum intervention concentration that did not affect cell viability. To clarify the protective effect of YQHX, H/R models of cardiomyocyte injury were intervened with YQHX. We found that compared with the control group, the release of LDH and CK in the H/R group was significantly higher in the primary cardiomyocytes. Interestingly, compared with the H/R group, the YQHX group showed significant inhibition of LDH release and CK activity (Figures 2(a) and 2(b)). Similar results were observed when using H9c2 cells. Compared with that in the control group, LDH release in the H/R group increased and significantly decreased after YQHX intervention (Figure 2(c)).

### 3.3. YQHX Decreases Bax/Bcl-2 and Caspase-3 in the H/R Model of Primary Cardiomyocytes

**Figure 3:**
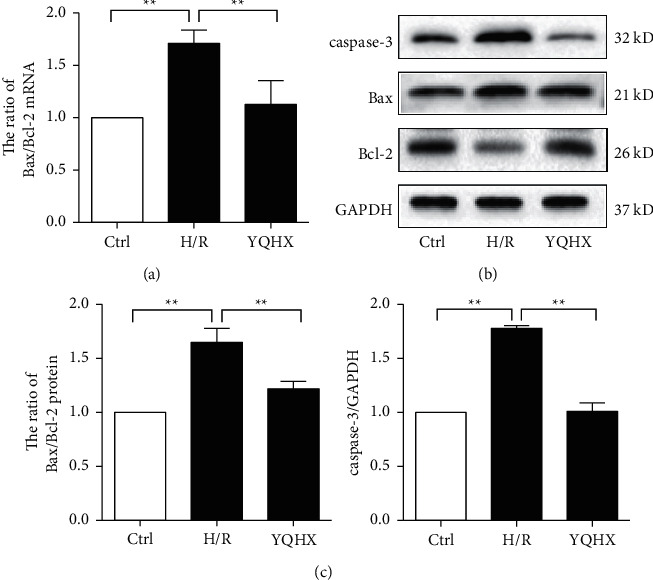
YQHX decreases Bax/Bcl-2 ratio and caspase-3 levels in the H/R model of primary cardiomyocytes. (a) mRNA expression of Bax and Bcl-2 was detected using qRT-PCR, and the Bax/Bcl-2 ratio was calculated. (b, c) Protein levels of Bax, Bcl-2, and caspase-3 were determined using western blot, and the Bax/Bcl-2 ratio was calculated. Data are expressed as mean ± SD of three independent experiments. ^*∗∗*^*p* < 0.01 was considered statistically significant.

The Bax/Bcl-2 mRNA ratio in the H/R group was significantly higher, compared with that in the control group and was significantly decreased after YQHX intervention (*p* < 0.05) (Figure 3(a)). The protein expression of Bax/Bcl-2 and caspase-3 was upregulated in the H/R group than in the control group. YQHX treatment significantly ameliorated caspase-3 and Bax/Bcl-2 expression (Figures 3(b) and 3(c)). These data suggested an inhibitory effect of YQHX in cardiomyocyte apoptosis.

### 3.4. YQHX Decreases Bax/Bcl-2 and Caspase-3 in the H/R Model of H9c2 Cells

**Figure 4:**
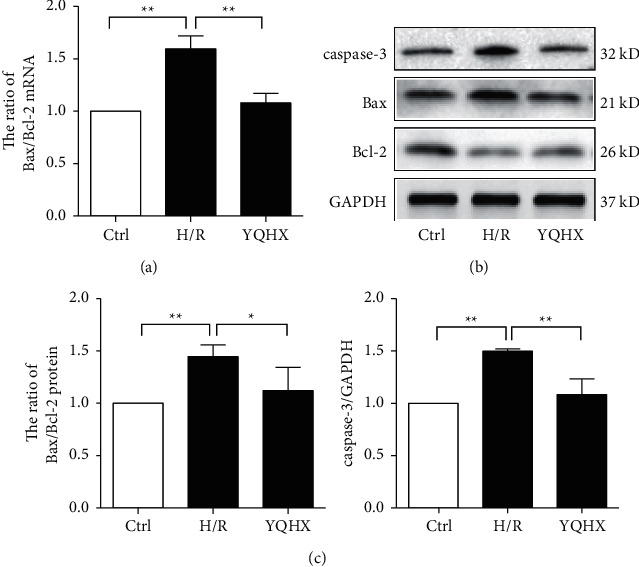
YQHX decreases Bax/Bcl-2 ratio and caspase-3 levels in the H/R model of H9c2 cells. (a) Bax and Bcl-2 mRNA levels were measured using qRT-PCR to calculate the Bax/Bcl-2 ratio. (b, c) Protein levels of Bax, Bcl-2, and caspase-3 were determined using western blot, and the Bax/Bcl-2 ratio was calculated. Data are expressed as mean ± SD of three independent experiments. ^*∗*^*p* < 0.05 and ^*∗∗*^*p* < 0.01 were considered statistically significant.

In addition to primary cardiomyocytes under H/R condition, damage and apoptosis were also evaluated in the H/R model of H9c2 cells. Bax/Bcl-2 and caspase-3 expression was significantly higher in the H/R group than in the control group (Figure 4). However, YQHX treatment significantly inhibited the increase in H/R-induced levels of Bax/Bcl-2 and caspase-3. The results were similar to those obtained for the primary cardiomyocytes.

### 3.5. YQHX Downregulated miR-1 Expression in the H/R Model of Cells

**Figure 5:**
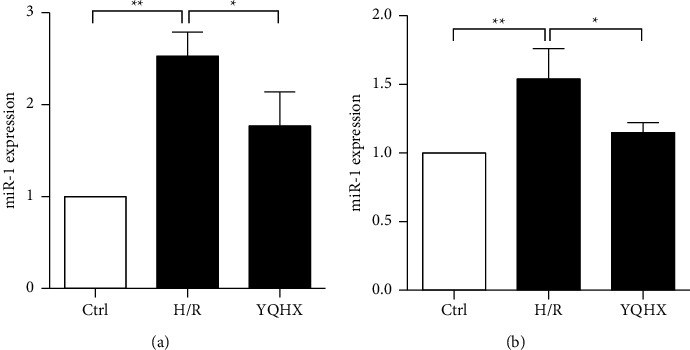
YQHX decreased miR-1 expression in the H/R model of cells. (a) miR-1 expression in primary cardiomyocytes was determined using qRT-PCR. (b) miR-1 expression in H9c2 cells was determined using qRT-PCR. Data are expressed as mean ± SD of three independent experiments. ^*∗*^*p* < 0.05 and ^*∗∗*^*p* < 0.01 were considered statistically significant.

To examine the apoptosis-related role of YQHX in the H/R model, the expression of miR-1 in primary cardiomyocytes and H9c2 was determined using qRT-PCR. We found that miR-1 was obviously upregulated in the H/R group than in the control group. However, compared with that in the H/R group, miR-1 was significantly decreased in the YQHX group (Figure 5, *p* < 0.05).

### 3.6. YQHX Inhibited Cardiomyocyte Apoptosis by Inhibiting miR-1 Levels

**Figure 6:**
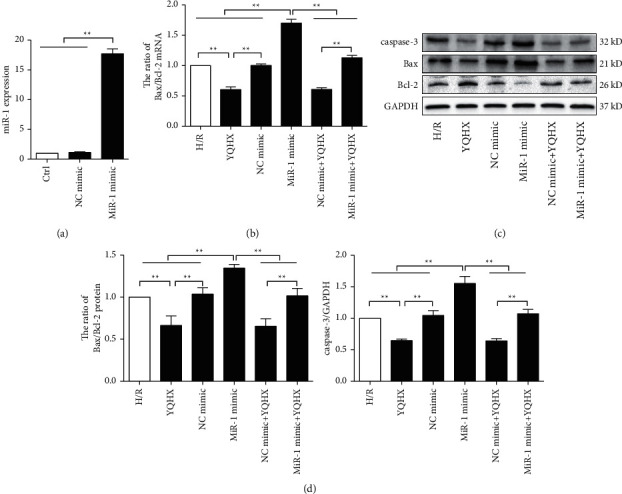
YQHX exhibited anticardiomyocyte apoptosis effects by inhibiting miR-1. (a) Transfection efficiency of miR-1 in H9c2 cells was determined using qRT-PCR. (b) MiR-1 mimics were transfected into H9c2 cells for 36 h before H/R modeling. Bax and Bcl-2 mRNA levels were using qRT-PCR, and the Bax/Bcl-2 ratio was calculated. (c, d) Protein levels of Bax, Bcl-2, and caspase-3 were determined using western blot, and the Bax/Bcl-2 ratio was calculated. Data are expressed as mean ± SD of three independent experiments. ^*∗∗*^*p* < 0.01 was considered statistically significant.

The above results indicated that the significantly increased expression of miR-1 in the H/R model correlated positively with myocardial injury and the apoptosis rate in cardiomyocytes. However, treatment with YQHX significantly decreased miR-1 expression and alleviated myocardial cell apoptosis and myocardial injury. Therefore, we speculated that YQHX might exert an anticardiomyocyte apoptosis role by regulating the expression of miR-1. To our hypothesis, H9c2 cells were transfected with miR-1 mimics. We found that this step significantly increased the miR-1 expression in the transfection group (Figure 6(a)). Moreover, Bax/Bcl-2 and caspase-3 levels were significantly increased in the miR-1 mimic group than the H/R group. Notably, YQHX intervention significantly decreased Bax/Bcl-2 and caspase-3 levels in the miR-1 mimic + YQHX group compared with that in miR-1 mimic group (Figures 6(b)–6(d)). These results indicated that the anticardiomyocyte apoptosis effect of YQHX was related to the inhibition of miR-1 levels.

## 4. Discussion

In this study, we showed that the release of LDH and CK activity was significantly increased in the H/R models of primary rat cardiomyocytes and H9c2. However, YQHX intervention significantly reduced the release of LDH and CK activity, thereby alleviating H/R-induced myocardial injury. Furthermore, YQHX reduced the Bax/Bcl-2 ratio and caspase-3 levels and downregulated miR-1 expression. These findings suggested that the anti-MIRI cardiomyocyte apoptosis effect of YQHX could be related to the decreased expression of miR-1, which may be a potential therapeutic target against MIRI.

In AMI, after the occluded blood vessel is reopened, various degrees of myocardial damage can affect the patients' quality of life and long-term prognosis [22]. During myocardial injury, the myocardial cell membrane is damaged and a large amount of LDH is released. In cardiomyocytes, CK, an important kinase involved in energy transport and ATP regeneration, mainly existing in the cytoplasm and mitochondria. Cell damage leads to an increase in CK activity [23]. In this study, an increase in LDH release and CK activity confirmed cardiomyocyte damage in H/R. However, intervention with YQHX alleviated these effects, thereby suggesting a protective effect of YQHX via a reduction of MIRI-induced myocardial injury.

Cardiomyocyte apoptosis is the main cause of myocardial injury in MIRI. Therefore, attenuating cardiomyocyte apoptosis has emerged as an important strategy to improve MIRI. Various apoptosis-related factors, including Bcl, Bax, and caspase-3, are involved in cardiomyocyte apoptosis. The protein levels of these factors are closely related to the degree of cardiomyocyte apoptosis [24]. In several studies, it has been reported that prescriptions for Qi-invigorating blood-activation (similar to the principle of traditional Chinese medicine used in this study) exhibit anticardiomyocyte apoptosis effects and improve heart function [25–27]. Here, we showed that YQHX reduced the levels of Bax/Bcl-2 and caspase-3 in H/R and, thereby, exhibited an antiapoptotic effect in cardiomyocytes. This is similar to the Qi-invigorating results, suggesting certain anticardiomyocyte apoptosis effects of YQHX. These effects were examined in both H9c2 cells and primary cardiomyocytes; therefore, the data are more comprehensive.

**Figure 7:**
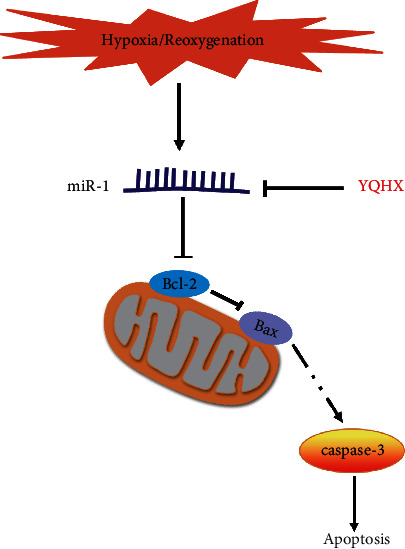
Schematic mechanisms of the antiapoptosis effects of YQHX.

miR-1 is highly expressed in the heart and plays an important regulatory role in MIRI during myocardial infarction [28, 29]. The regulatory role of miR-1 in cardiomyocyte apoptosis is closely related to the downregulation of Bcl-2, upregulation of caspase-3, and the aggravation of mitochondrial injury [30–33]. Reports on the intervention of traditional Chinese medicines or the use of monomer on miR-1 in MIRI are rare. For example, picroside which inhibits apoptosis in cardiomyocytes is also known to reduce miR-1 expression [34]. The active components of YQHX, including hydroxysafflower yellow A, astragaloside IV, paeoniflorin, and ginsenoside-Rb1 can inhibit cardiomyocyte apoptosis by downregulating miRNA-1 expression [20, 25, 35, 36]. Our findings suggested that miR-1 expression was increased under H/R conditions and could be significantly reduced after treatment with YQHX. Overexpression of miR-1 antagonized the antiapoptotic effect of YQHX. Overall, these results suggest that YQHX alleviates H/R-induced cardiomyocyte apoptosis by downregulating miR-1 (Figure 7).

### 4.1. Limitation

Our study had a few limitations. We mainly focused on in vitro experiments and did not extensively conduct in vivo studies. Also, we only explored the mechanism of the antiapoptosis effects of YQHX and did not study the mechanism of the antiapoptosis effects of the components in YQHX. Thus, in future studies, we will conduct in vivo experiments to observe the protective effects of YQHX in rat H/R injury and detect caspase activity and the Bax/Bcl-2 ratio to determine the extent of apoptosis. And, how the components in YQHX collectively regulate myocardial apoptosis through miR-1 will be further investigated in the future.

## 5. Conclusion

YQHX protects H9c2 cells and primary cardiomyocytes against H/R injury by downregulating miR-1. Moreover, it inhibits Bax/Bcl-2 expression and caspase-3 activation and alleviates cardiomyocyte apoptosis. These findings show the potential of YQHX as a novel strategy for the treatment of MIRI and related conditions in a clinical setting.

## Figures and Tables

**Table 1:** Primer sequences used for PCR.

	Forward	Reverse
miR-1	GCGCGTGGAATGTAAAGAAGT	AGTGCAGGGTCCGAGTATT
U6	CTCGCTTCGGCAGCAGCACA	AACGCTTCACGAATTTGCGT
Bax	AAGAAGCTGAGCGAGTGTCTC	AGTAGAAAAGGGCAACCACCC
Bcl-2	TCCTTCCAGCCTGAGAGCAACC	TGGACCACAGGTGGCACAGG
GAPDH	CTTCTCTTGTGACAAAGTGGACT	TTGAACTTGCCGTGGGTAGAG

## Data Availability

The datasets used and/or analyzed in the current study are available from the corresponding author upon reasonable request.
